# Simultaneous observation of intermittent locomotion of multiple fish by fine-scale spatiotemporal three-dimensional positioning

**DOI:** 10.1371/journal.pone.0201029

**Published:** 2018-07-19

**Authors:** Junichi Takagi, Kotaro Ichikawa, Nobuaki Arai, Yoshinori Miyamoto, Keiichi Uchida, Jun Shoji, Hiromichi Mitamura

**Affiliations:** 1 Graduate School of Informatics, Kyoto University, Kyoto, Japan; 2 CREST, Japan Science and Technology Agency, Saitama, Japan; 3 Field Science Education and Research Center, Kyoto University, Kyoto, Japan; 4 Department of Ocean Science, Faculty of Marine Science, Tokyo University of Marine Science and Technology, Tokyo, Japan; 5 Graduate School of Biosphere Science, Hiroshima University, Hiroshima, Japan; University of Western Australia, AUSTRALIA

## Abstract

Recent advances in biotelemetry techniques, especially positioning methods, have revealed the detailed behaviour and movement of aquatic organisms. Behavioural intermittence in animal locomotion, such as the Lévy walk, is a popular topic in the field of movement ecology. Previous attempts to describe intermittent locomotion quantitatively have been constrained by the spatial and temporal resolution possible with conventional biotelemetry systems. This study developed a fine-scale spatiotemporal three-dimensional positioning method using a new biotelemetry system with a positional precision of <10 cm and positioning interval of <10 s. Using this proposed positioning method, the intermittent stop-and-go locomotion of Siebold’s wrasses (*Pseudolabrus sieboldi*) was observed during travel from an unsuitable to a suitable location following displacement. The fish displayed behavioural intermittence in relocating to a suitable location. Initially, their movement halted for reorientation, after which they moved intermittently yet in a straight line to the suitable location. To test the positioning ability of the proposed method, data sets were resampled at intervals of 5, 10, 30, 60, and 300 s. Longer sampling intervals failed to identify reorientations and underestimated the number of stops, distance travelled, and speed. Overall, the results highlighted the adequacy and ability of the proposed positioning method to observe the intermittent locomotion of fish, such as stop-and-go behaviour, in a natural environment.

## Introduction

Biotelemetry techniques have been widely used in the past two decades to monitor the behaviour and movement of many aquatic organisms [1–3]. Acoustic positioning methods using ultrasonic transmitters and moored receivers have also advanced to meet the challenges of observing the exact positions of aquatic organisms, including fish, with accuracy of approximately 3−10 m based on a hyperbolic positioning technique (see [4–9]). These positioning methods have revealed the details of behaviour and movement of tagged individuals using positional information (e.g. homing behaviour [10–11] and movement patterns [12–13]). Behavioural intermittence (e.g. stops, or drastic changes in speed) is also a popular topic in movement ecology. Behavioural intermittence is significant to animal locomotion during activities (e.g. feeding, directed movement toward a nest, or habitat assessments) because sharp reorientations often follow the intermittence [14–15]. However, there are many challenges to tracking aquatic organisms at finer-scale temporal (5−10 s) and spatial (<1 m) resolutions. This is because conventional telemetry systems require a wider signal-transmitting interval (>30 s) to reduce collisions between received signals [16], and because the positioning accuracy of such systems is merely 3−10 m [4–9].

Recently, telemetry systems have been developed to enable the simultaneous tracking of multiple fish at higher temporal resolutions. For example, CDMA MAP technology was used to collect positional data on 22 fish at 15 s intervals with sub-metre accuracy [17]. A new biotelemetry system consisting of phase modulation-coded transmitters (so-called Gold code transmitters) and receivers, developed by Miyamoto et al. [18–19], can simultaneously identify ~75% of multiple coded signals at a transmitting interval of 1.28 s [20]. As the receiver has a sampling frequency of 1 MHz, the theoretical baseline positional resolution will be only 1.5 mm (dividing the underwater sound velocity 1,500 m•s^-1^ by 1,000,000 Hz) based on the time-difference-of-arrival (TDOA). This biotelemetry system should thus be capable of simultaneously observing the precise movement of multiple fish, a feat that conventional telemetry positioning systems with sub-metre accuracy have not achieved. However, the positioning performance of this new biotelemetry system has never been examined.

In this study, we propose a simultaneous positioning system with high spatiotemporal resolution using Gold code transmitters and receivers. In order to evaluate the positioning performance of the proposed system, multiple stationary transmitters and free-swimming fish (Siebold’s wrasse *Pseudolabrus sieboldi*) were simultaneously localised. Siebold’s wrasse is one of the most common species in rocky areas on the western coast of Japan [21]. Males demonstrate site fidelity during the spawning season (from late September to mid-November) [22]. Thus, we expected that they would exhibit homing behaviour to their original rocky area after being moved to an unsuitable place. Wrasses are well suited for studying the movement of fish using the developed positioning system as they travel in their natural environment from an unsuitable to a suitable place, i.e. the original rocky areas. Subsequently, the stop-and-go behavioural intermittence of the fish homing to their original location was observed.

## Materials and methods

### Biotelemetry system

Two types of Gold code transmitters and acoustic monitoring receivers (AQRM-1000, AquaSound Inc., Kobe, Japan; 64 mm in diameter and 300 mm in length), successors to the models developed by Miyamoto et al. [18–19], were used in this study. Gold code transmitters transmit signals consisting of three consecutive 2 ms short pulses coded by one of 32 Gold codes at a frequency of 62.5 kHz. Gold codes are single pseudo noise (PN) codes in pairs of M-sequences, which can decrease the false identification rate [23]. Pressure and temperature sensors were incorporated into the Gold code transmitters, allowing swimming depth and ambient temperature information to be encoded in the intervals between the 1^st^ and 2^nd^, then 2^nd^ and 3^rd^ pulses, respectively. The system accuracies were 0.5 m (pressure sensor), and 0.2°C (temperature sensor). Temperature data observed was not used for analysis in this study because it was unnecessary for evaluating the positioning performance. The acoustic monitoring receiver simultaneously identified multiple pulses from multiple transmitters simultaneously using cross-correlation values [20]. The receivers stored detection data in CSV format on an SD card. Data was then retrieved and downloaded for analysis. Receivers had a battery life of approximately two months with three D-size cells.

### Study area and deployed array

**Fig 1:**
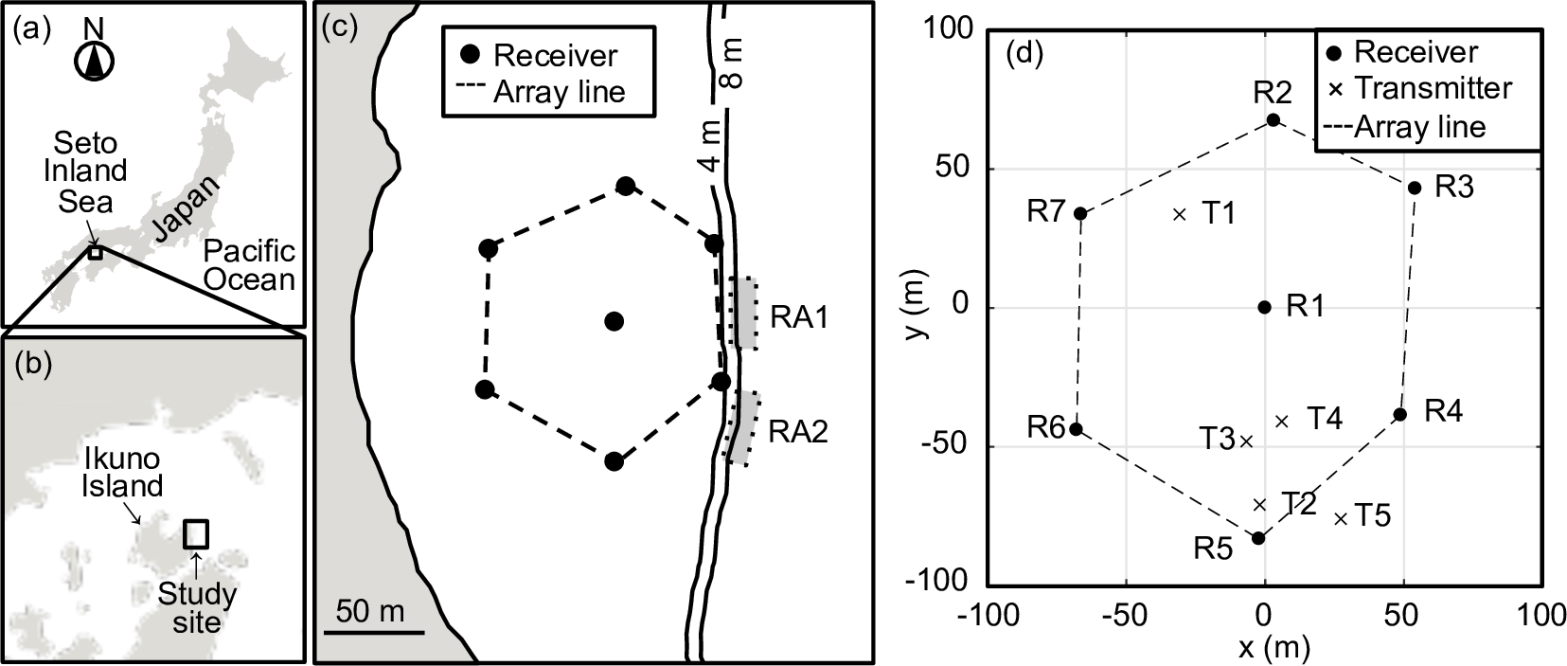
Acoustic array of seven receivers and transmitters for the experiment deployed off Ikuno Island, central Seto Inland Sea, southwestern Japan. **(a)–(c)** The array line represents the boundary between the outside and inside of the array. Vertical lines denoted by 4 m and 8 m represent the depth contour. There were two rocky areas (RA1 and RA2) on the east side of the array, where the Siebold's wrasses used as specimens in this study were caught. (d) Seven receivers (R) and five transmitters (T) fixed on the seafloor. Horizontal and vertical axes denote the relative coordinate axes, adjusted to the central receiver (R1) of the array.

Field experiments were conducted in a shallow sea area near the east coast of Ikuno Island (34°29'N, 132°92'E) in the Seto Inland Sea, Japan, in October 2015 (Fig 1). The depth of the seafloor in the area was separated into shallower (<4 m) and deeper (>8 m) levels by steep gradients (Fig 1C). The sea bottom of the area was covered with sand and mud. There were two rocky areas in the deeper area along the boundary between the two levels (Fig 1C). The tidal difference during the experiments was approximately 3.5 m.

An array of seven receivers with overlapping detection ranges was deployed on the seafloor in the study area (Fig 1D), covering an area of 13590.2 m^2^. Actual receiver locations were determined using a handheld GPS unit (Garmin eTrex 30J). A single Gold code transmitter (AQTD-600P, AquaSound Inc., Kobe, Japan; 27 mm diameter and 130 mm length) was placed on each receiver. The transmitters emitted signals at ~60 s intervals with a root mean square (RMS) sound pressure level (SPL) of 160 dB re 1 μPa at 1 m. The transmitter’s battery life was approximately one month using a single CR2 lithium cell. The sound speed was calculated using mutual signal detections between all receiver pairs. Receiver internal clocks were synchronised using calculated sound speed, determined receiver locations, and signal TDOAs. A conservative range (~75 m) between the receivers was used to increase mutual signal detections between receivers, although a preliminary experiment showed their detection range to be >100 m.

### Positioning method

The horizontal and vertical components of 3D positions were obtained separately. Horizontal positions were calculated using the proposed acoustic positioning method in this study, whereas vertical positions (swimming depth) were provided by a pressure sensor installed in the transmitters. 3D positioning was not feasible because the study site had little vertical range (~2–8 m) compared to its horizontal range (~75 m).

**Fig 2:**
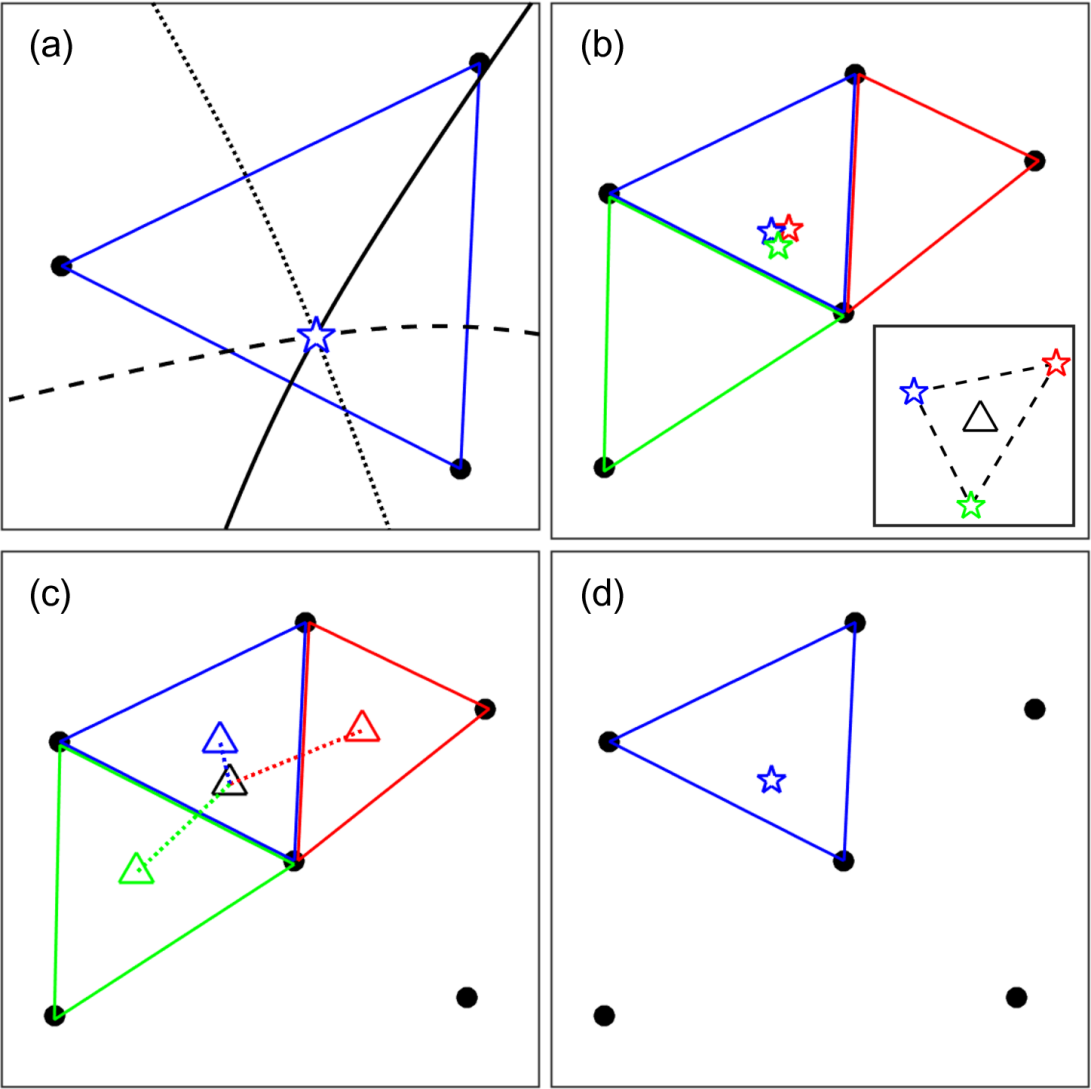
Example procedure of the proposed positioning method in this study. (a) A sound source position of a signal was horizontally estimated as an intersection (blue star) of three hyperbolae (bold, dashed, and dotted lines) by any subsets of close equilateral triangles. (b) When the signal was detected by five receivers forming three subsets (red, green, and blue triangles), three tentative estimates (red, green, and blue stars) were obtained by each subset. (c) Then, the distance between the centroid of three tentative estimates (small black triangle) and the three centroids of each subset (small red, green, and blue triangles) was calculated. (d) Finally, the estimated position by the blue triangle subset was selected for the final estimate using a criterion of the shortest distance.

When the three receivers detected a signal, the horizontal position of its sound source was estimated as an intersection of the three hyperbolae formed by signal TDOAs, the calculated sound speed, and the distance between the receivers for a final resolution of 1 mm (Fig 2A). Six subsets of close equilateral triangles were used to estimate positions: R1, R2, and R3; R1, R3, and R4; R1, R4, and R5; R1, R5, and R6; R1, R6, and R7; or R1, R7, and R2 (Fig 1D). When a signal was detected by the receivers that included two or more subsets of the close equilateral triangles, the position was estimated using every subset (Fig 2B). A single position was then selected using the shortest distance between the centroid of the estimated positions and the centroid of the close equilateral triangles used for positioning (Fig 2C and 2D), because shorter distances imply improved horizontal delusion of precision (HDOP) values. The HDOP represents the horizontal portion of the delusion of precision (DOP), which is universally used as a positioning precision index for GPS [24]. All positioning calculations were performed using relative coordinates modified from universal transverse Mercator (UTM) coordinates, the origin of which was adjusted to R1 (Fig 1D). Positioning and spatial analyses were performed in Matlab R2017a (The Math works, Natick, Ma, USA).

### Stationary test

The positioning performance of the proposed method was tested using stationary Gold code transmitters (AQPX-1040PT, AquaSound Inc., Kobe, Japan; 9.5 mm diameter, 36 mm length, 1.6 g weight in water) with a signal-transmitting interval of ~5.0 s and RMS SPL of 155 dB re 1 μPa at 1 m. Their battery life was approximately one week. Five Gold code transmitters were placed on the seafloor at five fixed points for 20 min. The seafloor depths of the fixed Gold code transmitter positions ranged from 2.5–2.8 m (Table 1). Two of them (T1 and T4) were placed near a centroid of triangulation, one (T2) near a vertex, one (T3) on a baseline, and the remaining one (T5) outside triangulation (Fig 1D).

**Table 1:** Summary of deployment information for the five transmitters employed during stationary testing.

Transmitter ID	Location	Installation depth (m)
T1	Near triangulation Centre	2.5
T2	Near vertex	2.5
T3	On baseline	2.7
T4	Near triangulation Centre	2.5
T5	Outside triangulation	2.8

The positional precision and probability of location were calculated, allowing both the spatial and temporal resolution of the proposed positioning method to be evaluated. The positioning precision was defined as the mode distance between each estimate and the centroid of the estimates because the distribution form of the distance was right-skewed. The mean±standard deviation (sd) and 95-percentile distances of each estimate from the centroid of the estimates were also calculated. Furthermore, the directional distribution (95% deviational ellipse) of the estimates was displayed for visual comprehension. The probability of location was defined as the proportion (%) of the number of estimates to the number of actual transmissions made by each Gold code transmitter. The positional accuracy (i.e. the distance between the estimated and actual location) was not evaluated in this study because the predicted positional precision was significantly smaller than the GPS accuracy (normally a few metres).

A generalised linear mixed model (GLMM) using the gamma distribution with the log link function was employed to examine whether the location of the transmitter (near the triangulation centre, near the vertex, on the baseline, and outside triangulation) affected positional precision. The model included the location of the transmitter as a fixed effects factor and the transmitter IDs as a random effects factor. The model was fitted using the glmer function in the lme4 package for R ver. 3.1.3 [25].

### Free-swimming fish test

Positioning performance was also tested using Siebold’s wrasse as free-swimming fish specimens. Seven male Siebold’s wrasses (N = 7, total length (TL): 21.5±0.9 cm (mean±sd), body weight (BW): 150.1±15.2 g) (Table 2) were captured by fishing with a baited hook and line in two rocky areas (Fig 1C) between 23 to 26 October 2015. One of the seven fish (F1) was caught in rocky area 1, and the remaining six (F2−7) in rocky area 2. Specimens were kept in a round tank (~1280 mm diameter, 815 mm height, 1000 litre volume) for 2–5 days until the experiment. The tank was filled with ~600 litre fresh seawater flowing of ~10 litre•min^-1^. Gold code transmitters (AQPX-1040PT, AquaSound Inc., Kobe, Japan) with signal transmitting intervals of ~5.0 s were surgically inserted into the abdominal cavities of the fish under anaesthesia induced with 0.1% 2-phenoxyethanol. The fish were placed between rubber sponges in baths of fresh bubbling seawater throughout the procedure. After the procedure, they were kept in fresh seawater until they came out of the anaesthesia.

**Table 2:** Fish information of the seven tagged Siebold’s wrasses.

Fish ID	Body Weight (g)	Total Length (cm)
F1	173	22.4
F2	152	21.5
F3	135	21.1
F4	127	20.0
F5	152	22.3
F6	159	22.2
F7	153	21.1
Mean ± sd	150.1 ± 15.2	21.5 ± 0.9

The tagged fish were then placed in an upside down transparent container with small holes and kept on the sea bottom (3.5 m depth) at a release point inside the array approximately 87 m and 132 m away from rocky areas 1 and 2 (Fig 1C), respectively. The release point was on a flat sea bottom of muddy sediment. As the tagged fish inhabited and had been caught in rocky areas, the release point was suspected to be an unsuitable environment for them. After 35 min of acclimation, the container was slowly opened and the tagged fish were simultaneously released at 14:56 on 28 October 2015. Their 3D positions were monitored until 9:00 on 30 October. Whether the tagged fish homed to the rocky area was determined using an additional receiver deployed at each rocky area the next day. The additional two receivers were not used for positioning.

Outliers were removed from raw data obtained by the proposed positioning method using speed limitation. We conservatively set 5 TL•s^-1^ as the maximum swimming speed in this study due to the cruising speed (generally less than 2–3 body length•s^-1^). Burst swimming was not considered because, in this study, the target movement was relatively long-term movement while traveling. If the speed between two consecutive raw data points exceeded 5 TL•s^-1^, the latter point was removed. Removed data comprised 6.8±8.8% of the raw data (N = 7, range: 0–19.5% or 0–335 data points). To test the positioning performance of the proposed method for locating free-swimming fish following the same method as for the stationary test, the probabilities of location and positioning intervals of the seven tagged fish within the array were examined. The positioning interval was defined as the mean value of intervals between all consecutive pairs of data points.

**Fig 3:**
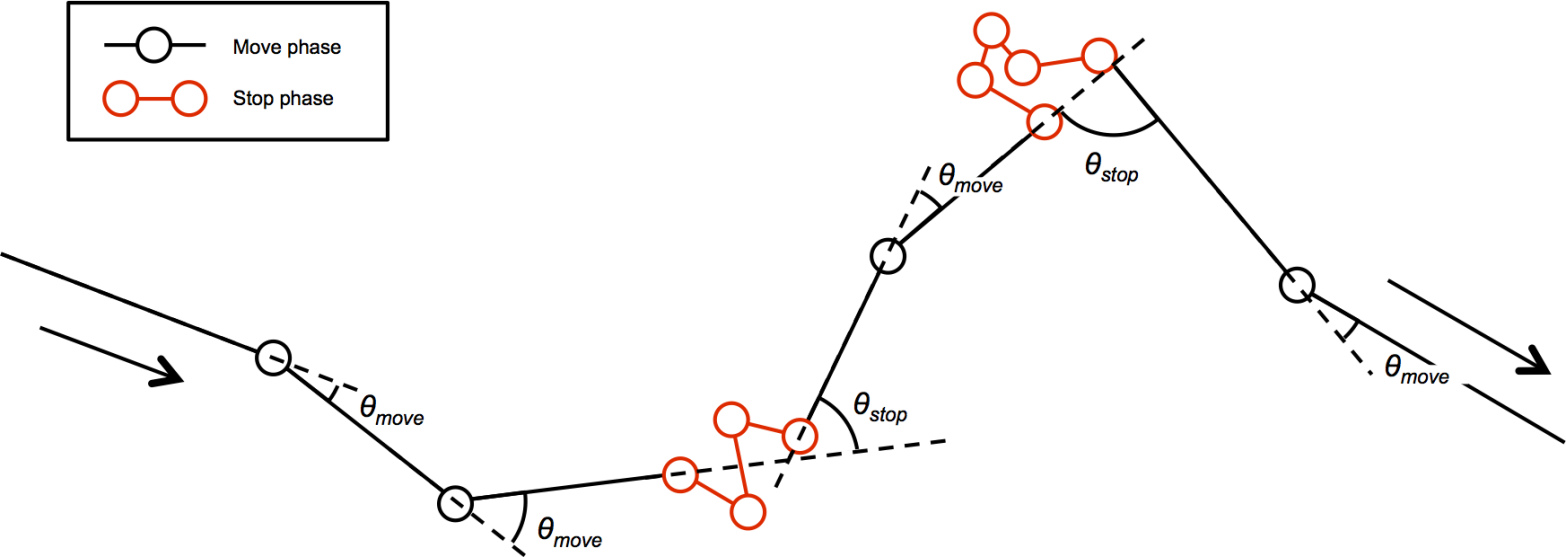
Schematic diagram of turn angles in a ‘move’ phase (*θ*_*move*_) and ‘stop’ phase (*θ*_*stop*_).

To examine the intermittent locomotion of fish travelling from an unsuitable to a suitable area, their locomotion modes were categorised into two types: ‘stop’ and ‘move’. First, routes between the release point and rocky areas were extracted using a swimming depth threshold of <4 m. Movement at night [30 min after sunset (17:52) to 30 min before sunrise (5:57)] was omitted from further analyses because Siebold’s wrasse follow the diurnal habits common to labrid fish and do not travel distances at night (e.g. [26–27]). Route data was spatially interpolated every 5 s if there were zero or one missing values between each data step (a temporal interval between two consecutive data points <~10 s). If the distance between two consecutive estimates was ≤0.64 m, the tagged fish was categorized in the ‘stop’ mode at both points. A ‘stop’ phase was defined as sequential ‘stop’ modes (Fig 3). Conversely, if the distance was >0.64 m, the tagged fish was categorized in the ‘move’ mode at the latter point. A ‘move’ phase was defined as sequential ‘move’ modes (Fig 3). Missing values were ignored in distance calculations. The 0.64 m threshold was defined as 2× the maximum 95-percentile distance from the stationary test (0.32 m; see Table 3). Turn angles in the two phases were then calculated. The turn angle in ‘move’ phases was defined as the difference in destinations between any set of three sequential estimates (-180°<θ≤180°; see Fig 3) whereas the turn angle of ‘stop’ phases was defined as the difference between the final destination of the preceding ‘move’ phase and the first destination of the following ‘move’ phase (-180°<θ≤180°; see Fig 3). Turn angles in ‘stop’ phases were categorised into two types, first-half turns and second-half turns, according to our hypothesis that the tagged fish would display movement with angled turns during the first half of their return based on the documented searching behaviour of homing fish (e.g. [11] and [28]). If the tagged fish stayed overnight in their travels, movement until 30 min after sunset and from 30 min before sunrise were categorised into the first half and the second half, respectively. If the tagged fish finished traveling within the release day, the first and second halves were separated by the traveling period. Turn angles were calculated only when all time step data was available without missing values. Circular statistical analyses were performed using functions in the circular package for R ver. 3.1.3 [25].

**Table 3:** Summary of the positioning results of the five transmitters employed during stationary testing.

Transmitter ID	Depth provided by pressure censor (m)	Detection rate (%)	Probability of location (%)	Distance (m) each estimate − centroid of estimates		
				Mode	Mean ± sd	95%
T1	2.5 ± 0.02	95.7 ± 5.0	96.0	0.012	0.081 ± 0.049	0.195
T2	2.5 ± 0.06	87.8 ± 14.9	80.6	0.093	0.111 ± 0.058	0.251
T3	2.7 ± 0.05	79.0 ± 18.5	60.5	0.037	0.110 ± 0.081	0.322
T4	2.5 ± 0.05	92.6 ± 10.9	90.7	0.057	0.069 ± 0.027	0.136
T5	2.8 ± 0.06	90.8 ± 8.9	90.4	0.080	0.115 ± 0.063	0.255

To show the spatiotemporal resolution of the proposed method, the number of ‘stop’ phases, distance travelled, speed, and turn angles were compared between five resampled data sets. Routes were also interpolated at 10, 30, 60, and 300 s intervals using the manner previously described. The distance travelled was calculated as the cumulative distance from ‘move’ phases and the distance between first and last point in ‘stop’ phases. Speed was calculated only where there were no missing values between any two consecutive interpolated data points in the ‘move’ phase. The speed distribution in each sampling interval (5, 10, 30, 60, and 300 s) was estimated using a kernel density estimation. Turn angles during the ‘move’ phase, first half of the ‘stop’ phase, and second half of the ‘stop’ phase at each sampling interval were examined using a Rayleigh test [29] to determine whether there was a uniform distribution. If there was not a uniform distribution, a V-test [29] was conducted to determine whether it was concentrated around 0°.

### Ethics statement

The Director-General of the Hiroshima Prefectural Agriculture, Forestry, and Fisheries Station issued permission for fish collection for this study around Ikuno Island. All procedures including the sampling protocol and the tagging surgery in this study were approved by the Animal Research Committee of Kyoto University (permit number: Inf-K15003).

## Results

### Stationary test

**Fig 4:**
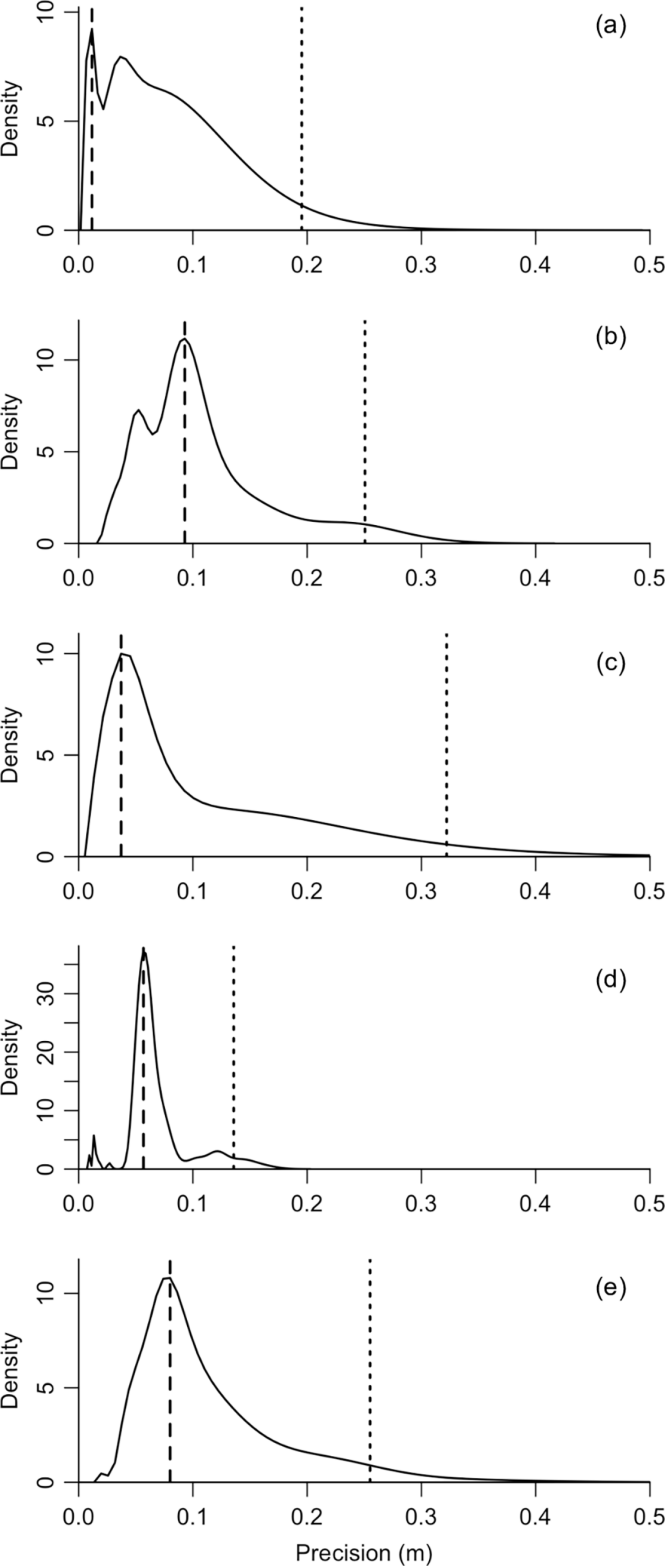
Kernel density estimation of the distance between the centroid of all estimates and each estimate of the five stationary transmitters. The distribution of transmitters 1–5 is represented in (a)–(e), respectively. The dashed line indicates the mode value, and the dotted line indicates the 95-percentile value.

**Fig 5:**
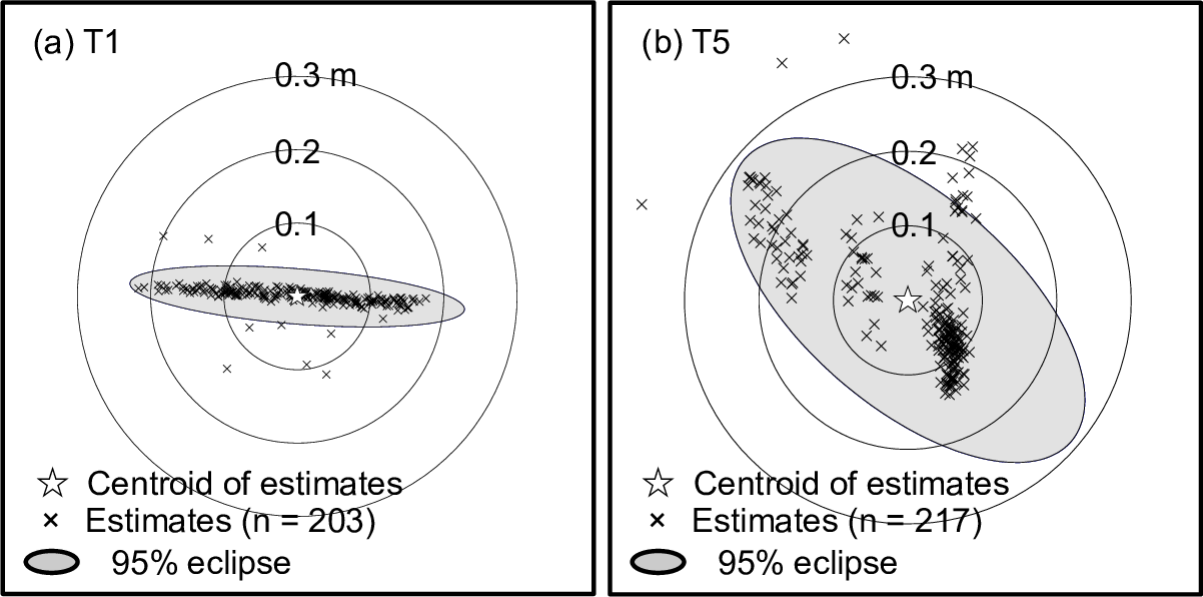
Stationary test results. (a) 2D estimates with their centroid, and 95% eclipse for the deployment of T1 near a centroid of triangulation, and (b) T5 outside triangulation.

The positional precision was 0.056±0.033 m (N = 5; range 0.012−0.093 m; Fig 4, Fig 5 and Table 3) and probability of location was 83.6±14.0% (N = 5, range 60.5−96.0%; Table 3). The estimated distribution of the distance between each estimate and the centroid of estimates was right-skewed in all transmitters (Fig 4). The 95-percentile of distance was 0.232±0.070 m (N = 5, range 0.136−0.322 m; Fig 4 and Table 3). The pressure sensor in the transmitters provided accurate depth information within a strict range, enabling high spatial resolution 3D positioning (Table 1 and Table 3). The results from the GLMM indicated that the positional precision of transmitters installed near centroid was higher than that of transmitters installed at other places (Table 4).

**Table 4:** Generalised linear mixed model for positional precision. Model coefficients for fixed effects are presented.

Explanatory variables	Coefficients	Standard error	t-value	p-value
(Intercept)	-2.59014	0.02654	-97.577	***
Near centroid^a^				
On baseline	0.38023	0.05499	6.915	***
Outside triangulation	0.42760	0.04702	9.093	***
Near vertex	0.38738	0.04812	8.051	***

^a^ Standard category

*** *p* < 0.001

### Free-swimming fish test

**Fig 6:**
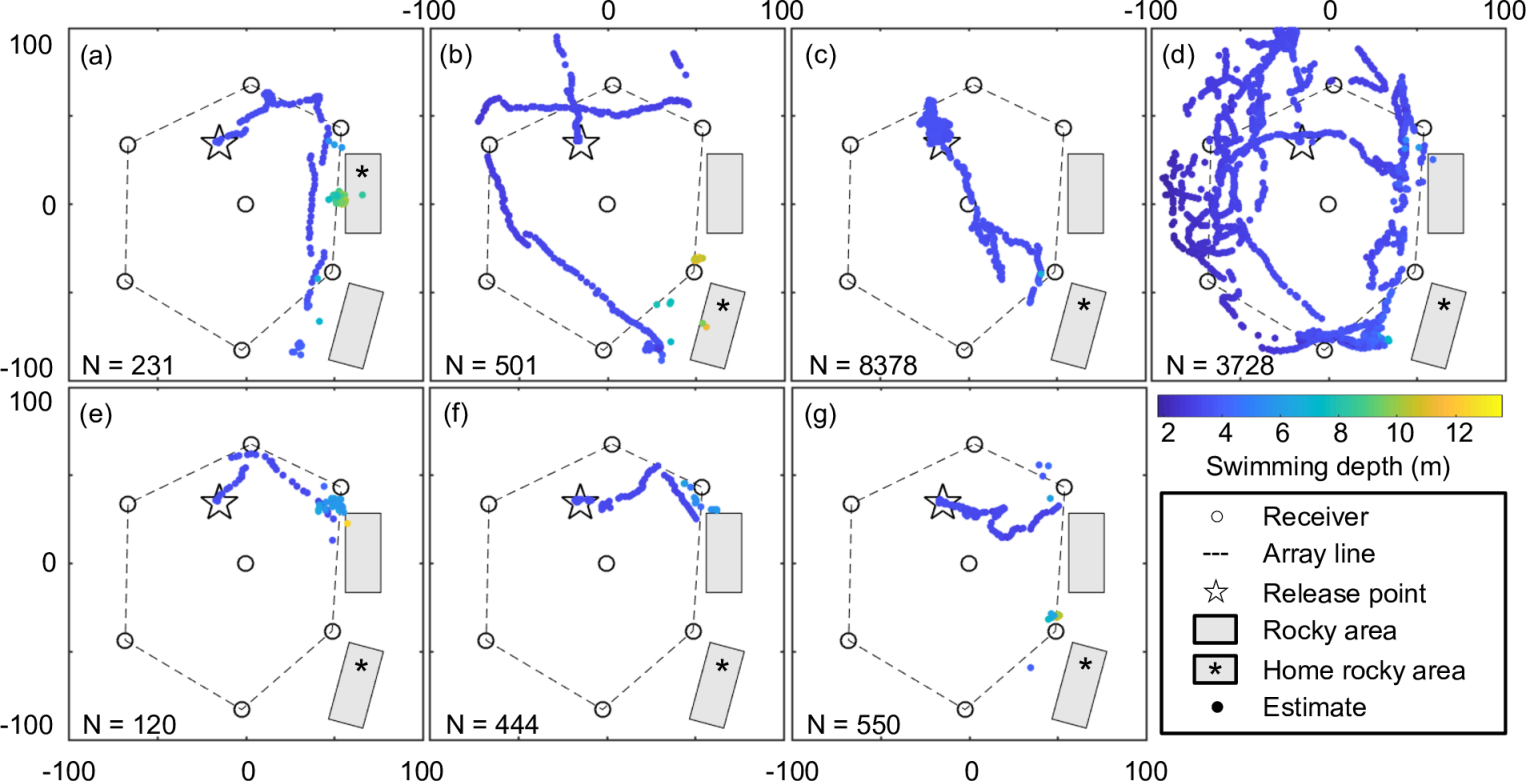
3D estimates of seven tagged Siebold’s wrasses (F1−7) during the monitoring period. Estimates for F1−7 are shown in (a)−(g), respectively, with colour variations indicating swimming depths.

The tagged fish stayed within the 13590.19 m^2^ array for 7574.3±17419.3 s (N = 7, range 280.1−47023.9 s; see Table 5), during which 1071±2424 estimates (N = 7, range 27−6558 estimates; see Table 5) were obtained. Their probability of location was 69.1±15.3% (N = 7, range 46.6−89.4%; see Table 5), and the positioning interval was 7.4±1.7 s (N = 7, range 5.6−10.4 s; see Table 5). Their swimming depth within the array averaged ~3 m, suggesting they remained on the seafloor during both ‘stop’ and ‘move’ phases at least in the shallower zone of the study area (Table 5 and Fig 6).

**Table 5:** Positioning results within the array of the seven tagged Siebold’s wrasses.

Fish ID	Residence time (s)	Number of estimates	Probability of location (%)	Mean positioning interval (s)	Swimming depth^a^ (m)
F1	461.4	58	59.8	8.0	3.5 ± 0.1
F2	521.9	93	89.4	5.6	3.4 ± 0.1
F3	47023.9	6558	69.2	7.2	3.6 ± 0.1
F4	280.1	27	46.6	10.4	3.4 ± 0.1
F5	320.1	38	57.6	8.4	3.4 ± 0.1
F6	1690.1	270	79.9	6.3	3.5 ± 0.1
F7	2723.0	458	81.2	5.9	3.3 ± 0.1
Mean ± sd	7574.3 ± 17419.3	1071 ± 2424	69.1 ± 15.3	7.4 ± 1.7	-

^a^ Represented as mean±standard deviation

All tagged fish returned to suitable areas (original rocky areas) from the unsuitable release area (a muddy flat area) before the following morning. Four of the seven tagged fish (F1, F5, F6, and F7) moved east and settled around the rocky areas before sunset on the release day, and a single tagged fish (F1) homed to its original location (Fig 6A, 6E, 6F and 6G). One tagged fish (F2) moved toward the north immediately following release, then turned clockwise and reached home before sunset by traveling around the array in a ‘Z’ pattern (Fig 6B). The remaining two tagged fish (F3 and F4) stayed around the array overnight: one (F3) settled near the release point, whereas the other (F4) settled at a northern point in the array after wandering the northern and western sides of the array until sunset. The next morning after sunrise, F3 and F4 moved toward the rocky areas, and F3 homed to its original location (Fig 6C and 6D).

Intermittent stop-and-go locomotion was observed in the routes of the seven tagged fish. Using 5 s sampling intervals, the tagged fish stopped 11.3±10.3 times (N = 7, range 1−30 times) on routes lasting 17851.4±27545.6 s (N = 7, range 275−58545 s; Table 6) excluding nights. The duration of the ‘stop’ phase was 277.6±653.5 s and the median duration was 30.0 s (N = 79, range 5−3555 s). The ‘move’ phase turn angle distribution at 5 s sampling intervals was significantly concentrated around 0° (V-test, mean vector = 0°, *W* = 0.72, p<0.001; Table 7). The first half ‘stop’ phase distribution was not significantly concentrated in any direction (Rayleigh test, *W* = 0.14, p>0.05; Table 7); conversely, the second half distribution was significantly concentrated around 0° (V-test, mean vector = 0°, *W* = 0.61, p<0.001; Table 7).

**Table 6:** Duration and distance travelled in five resampling intervals of movement routes of seven tagged Siebold's wrasses.

Fish ID	Duration^b^ (s)	Distance travelled (m)				
		5 s	10 s	30 s	60 s	300 s
F1	825	229.8	219.7	198.7	163.7	60.4
F2	3050	494.4	492.2	469.8	455.0	407.8
F3^a^	15045	288.6	279.3	251.1	222.6	146.2
F4^a^	14250	1817.5	1808.4	1759.6	1666.5	1188.5
F5	275	100.6	98.0	86.2	78.5	n/a
F6	1795	106.9	100.6	89.6	68.9	16.9
F7	2720	117.7	116.4	96.7	87.8	62.7

^a^ Without nights (17:52−5:57 the next day).

^b^ In 5 s resampling interval.

**Table 7:** Summary of turn angles in ‘move’ mode, and first and second halves of ‘stop’ mode at six sampling intervals.

Phase	Sampling interval (s)	Number of data	Test statistics^a^	p-value
Move	5	1373	V, *W* = 0.72	<0.001
	10	1120	V, *W* = 0.50	<0.001
	30	567	V, *W* = 0.34	<0.001
	60	381	V, *W* = 0.28	<0.001
	300	179	V, *W* = 0.22	<0.001
Stop in first half	5	57	R, *W* = 0.14	>0.05
	10	47	R, *W* = 0.29	>0.05
	30	29	R, *W* = 0.06	>0.05
	60	21	R, *W* = 0.12	>0.05
	300	17	R, *W* = 0.36	>0.05
Stop in second half	5	24	V, *W* = 0.61	<0.001
	10	19	R, *W* = 0.14	>0.05
	30	8	R, *W* = 0.55	>0.05
	60	6	R, *W* = 0.16	>0.05
	300	2	n/a	-

^a^ n/a indicates that there were too few data points to test. The V-test (V) with a mean vector of 0° was used when a null hypothesis of uniform distribution was rejected by the Rayleigh test (R).

**Fig 7:**
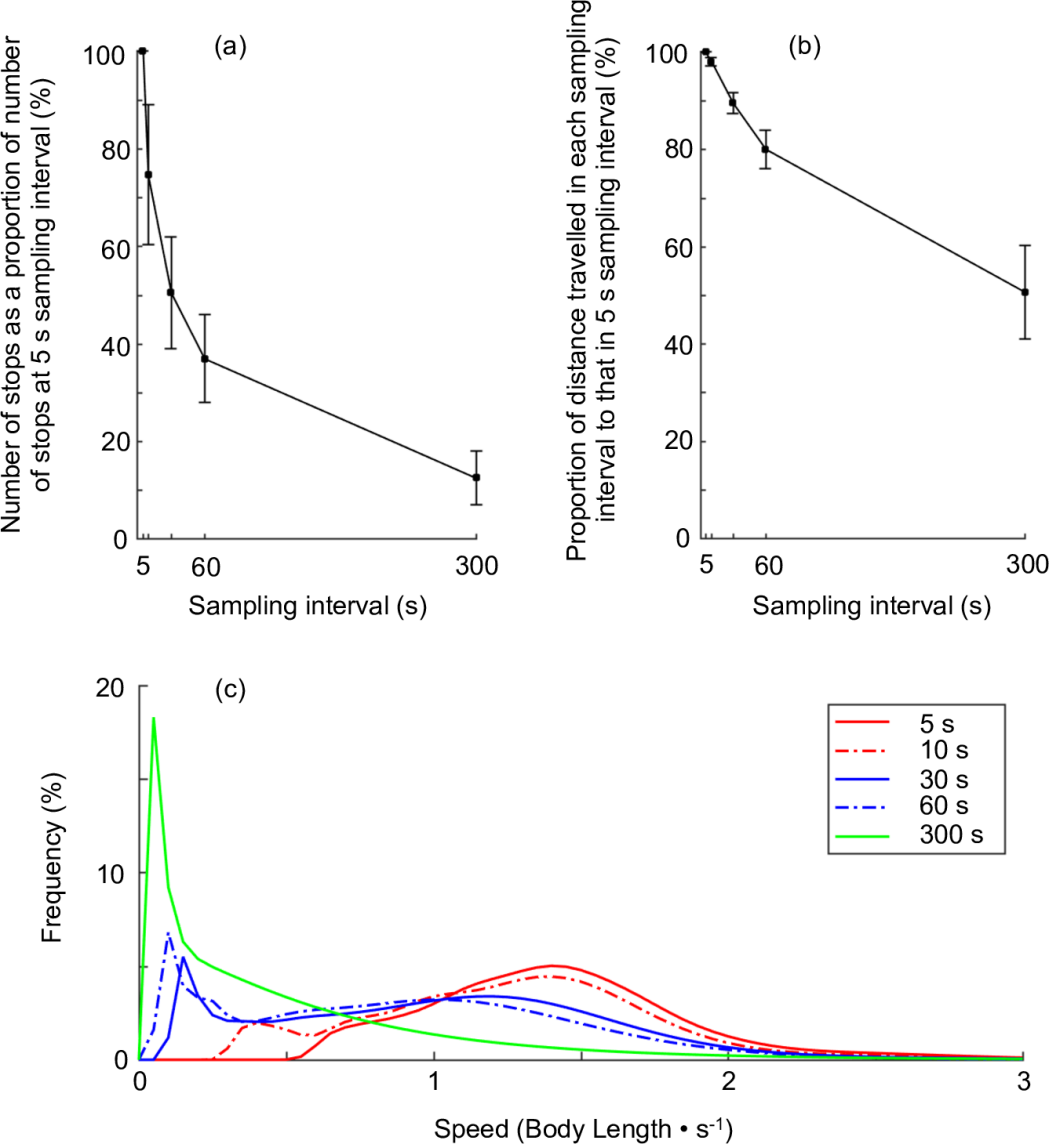
Comparison of stop numbers, distance travelled, and speed of tagged Siebold’s wrasse. (a) Number of ‘stop’ phases and (b) distance travelled as a proportion of that at 5 s sampling intervals for the seven tagged Siebold’s wrasse in five resampling interval lengths (5, 10, 30, 60 and 300 s). The error bar indicates the standard mean error. (c) Kernel density estimation of movement speed only during ‘move’ phase in the five resampling interval lengths. The speed was standardised (divided by the total length of each fish).

Increasing the sampling interval decreased the number of stop phases (Fig 7A) and also decreased the distance travelled (Table 6 and Fig 7B). The speed distribution in the ‘move’ phase became gradually right-skewed when increasing the sampling interval (Fig 7C). The turn angle distribution of the ‘move’ phase was significantly concentrated around 0° at all six sampling intervals (V-test, mean vector = 0°; see Table 7), while that of the first half of the ‘stop’ phase was not significantly concentrated in any direction with all six sampling intervals (Rayleigh test, p>0.05; see Table 7). In the second half of the ‘stop’ phase, there was no significant concentration in any direction in the turn angles sampled at 10, 30, and 60 s intervals (Rayleigh test, p>0.05; see Table 7) although there was less data with increased sampling intervals. At 5 s intervals, the turn angle distribution in the second half of the ‘stop’ phase had significant concentration around 0° (V-test, mean vector = 0°, *W* = 0.61, p<0.001; see Table 7).

## Discussion

### Stationary test

The proposed positioning system using Gold code transmitters and receivers positioned multiple stationary transmitters simultaneously and in 3D with high-precision spatial resolution (<10 cm) and a high probability of location (average >80%). The results demonstrate that the system is capable of simultaneously observing the movement of multiple aquatic animals at a high spatiotemporal resolution. The results from the GLMM show that the positional precision near the centroid of triangulation was significantly higher than that of other installation sites (near the vertex, on the baseline, and outside triangulation) although fewer transmitters were deployed during the stationary test (Table 5). This result is generally known and has been demonstrated in previous studies of positioning methods using the TDOA concept (e.g. [6–7]). However, the positional precision of five installation sites was considerably high. Even at the 95% point, the distance from the centroid of estimates was only 0.322 m (Table 3 and Fig 4). One transmitter (T3) had a relatively lower probability of location than those of the remaining four transmitters. This may have been caused by lower detection rates directly linked to poor mutual detection in three receivers (Table 3). Multiple non-detection and/or false-alarm factors should be considered [30]: differences in bathymetry or obstacles between transmitters and receivers (see [31]); environmental noise from motors, snapping shrimp, and other taxa (see [32]); and sea surface conditions (see [33]). Although signal collisions influenced detection rates [34], signals in the presented biotelemetry system rarely collided due to their short (2 ms) pulse duration [19]. This is one of the major advantages of the developed system. The lower detection rate may also be explained by the differences between the transmitters and/or the installation sites. The ultimate cause for this lower detection rate, however, remains unidentified.

Receiver array geometry and other environmental factors can decrease detection rates [6] and affect positioning performance. Furthermore, biotelemetry has been performed in various waters, such as shallow seas, deep seas, narrow rivers or channels, small ponds, and large lakes [2], where environmental factors affect the propagation of ultrasonic waves [28], resulting in the deterioration of positional precision and location probability. Receivers were deployed to form multiple equilateral triangles, and only equilateral triangulations were used for positioning with the proposed method. Acute- or obtuse-angled triangulation can mathematically deteriorate positional precision due to the use of hyperbolic curves during TDOA positioning [7]. It is necessary to deploy receiver arrays that are mathematically suitable for calculating as many locations as possible. The influence of environmental factors in the proposed biotelemetry system shall be investigated in future studies.

### Free-swimming fish test

Seven tagged Siebold’s wrasses were simultaneously positioned at high temporal resolution, providing precise data on their movement routes and behavioural intermittence. Intermittent stop-and-go locomotion was observed as the fish travelled from an unsuitable to a suitable area after their displacement. To the authors’ knowledge, this study was the first report to demonstrate the homing ability of Siebold’s wrasse. At a sampling interval of 5 s, turn angles in the ‘move’ and the second half of the ‘stop’ phase were significantly concentrated at 0°; conversely, those in the first half of the ‘stop’ phase were not concentrated in any direction (Table 7). This implies that the tagged fish tended to change direction after stopping with relatively straight movement during the first half, and travelled in a relatively straight direction during the second half. Thus, the stop-and-go behavioural intermittence of Siebold’s wrasse observed in this study appeared linked to reorientation during the first half of traveling from an unsuitable to a suitable area. Animal movement can be understood as scanning and reorientation sequences such as the saltatory search [35] or Lévy patterns [14–15]. Therefore, the tagged fish may have searched for the direction leading to a suitable place (a rocky area, as their familiar location), by scanning for environmental cues using sensory organs. It is possible the tagged fish used an olfactory cue from their habitat, similar to salmonids [36–37]. For instance, black rockfish *Sebastes cheni* perform back-and-forth movement in the direction of the current (intermittent locomotion with 180° turns) to detect their homeward direction using olfactory cues [11]. The tagged fish may have sensed an olfactory cue related to the current direction, for instance, an odour of conspecific females from their original location due to the spawning season. They may have also sensed other cues such as bathymetric gradients, including changes in light, temperature, and pressure with depth (see [38]). Although it is ultimately unknown what the tagged fish did during their first half ‘stop’ phases, this study demonstrated that the proposed positioning method is capable of observing the intermittent locomotion of free-swimming fish in their natural environment.

It was confirmed that the proposed temporally fine-scale positioning method enables observations of the stop-and-go travels undertaken by tagged fish. The number of observed ‘stop’ phases decreased as the resampling interval increased (Fig 7A). For example, relative to the original 5 s sampling interval, only 75% of ‘stop’ phases were observed at 10 s intervals. Although the same result might have obtained were the sampling interval less than 5 s, the results show that there were numerous undetected ‘stop’ phases when longer intervals were used. However, at a sampling interval of 5 s, it was observed that the tagged fish tended to reorient after stopping with straight movement during the first half, and tended to travel in a straight direction thereafter during the second half. This was not observed at >10 s sampling intervals. It was thus the positioning method, with fine-scale temporal resolution, that enabled the observation of stop-and-go travels of tagged fish and the elucidation of their straight movement and reorientation after stopping. Furthermore, the distance travelled was underestimated as resampling intervals increased (Fig 7B). Although the distance travelled tends to be overestimated at high frequency sampling intervals due to the fine scale of resolution, especially with GPS [39], it is suspected that the distance travelled at 5 s sampling intervals was not significantly overestimated because the step length while stopped was not considered, and this often leads to overestimations. The speed during ‘move’ phases was also underestimated as the sampling interval lengthened (Fig 7C). It should be considered that a precise sampling interval can never perfectly describe whole movement of tagged fish; nevertheless, it was the case that ‘stop’ phases related to reorientations were observed at precise (i.e. 5 s) sampling intervals, and not observed at >10 s sampling intervals.

Within the array, the probability of location of the tagged fish was 69.1±15.3%, which was relatively low compared to that of the stationary test (83.6±14.0%). The positioning interval, however, was 7.4±1.7 s, giving an estimate of tagged fish locations at least once every ~10 s. Conventional biotelemetry systems typically set their signal transmitting interval between 30–120 s to minimize collisions (see [40]); for example, during the simultaneous observation of moving routes using a conventional biotelemetry system, Mitamura et al. obtained positional data sets of the homing behaviour of four black rockfish (*Sebastes cheni*) at time intervals of 60 s or greater [11]. CDMA MAP technology, which has simultaneous positioning capabilities, was able to localise ~75% of transmissions at 15 s intervals, such that there was an estimate every 20 s on average [17]. Compared to these positional intervals, the proposed positioning method has the advantage of frequent monitoring in movement routes using positional data. Moreover, the biotelemetry system used in this study could potentially provide more temporally precise movement observations if the signal-transmitting interval were shortened (i.e. 1.0 or 2.0 s); however, the interval was set to ~5.0 s in this experiment. The utilized biotelemetry system is capable of simultaneously identifying multiple high-temporal resolution (<2 s) signals transmitted at 1.28 s intervals [20], such that positions can be obtained at a higher temporal resolution than 10 s. These temporally precise positions can provide more detailed movement data than the results shown in this study.

This study developed a positioning method capable of simultaneously pinpointing multiple fish with fine-scale data both in space and time using a new telemetry system that was validated by providing precise 3D positions of seven Siebold's wrasses. Those spatiotemporally fine-scale positions allowed intermittent locomotion to be observed, and revealed that the tagged Siebold’s wrasses tended to reorient after ‘stop’ phases during the first half of their voyage. This method will provide insights into unknown aquatic animal behaviour by providing more detailed movement observations in the future.

## Supporting information

S1 File Estimates of transmitters employed in stationary test. (CSV)

S2 File Estimates of Siebolt’s wrasses in free-swimming fish test. (CSV)
